# Evaluation of the malaria reporting system supported by the District Health Information System 2 in Solomon Islands

**DOI:** 10.1186/s12936-020-03442-y

**Published:** 2020-10-17

**Authors:** Kinley Wangdi, Haribondu Sarma, John Leaburi, Emma McBryde, Archie C. A. Clements

**Affiliations:** 1 Department of Global Health, Research School of Population Health, College of Health and Medicine, The Australian National University, 62 Mills Road, Canberra, ACT 2601 Australia; 2 National Centre of Epidemiology and Population Health, Research School of Population Health, College of Health and Medicine, The Australian National University, Canberra, Australia; 3 National Vector Borne Disease Control Programme, Ministry of Health and Medical Services, Honiara, Solomon Islands; 4 Australian Institute of Tropical Health & Medicine, Centre for Biosecurity in Tropical Infectious Diseases, James Cooks University, Townsville, Australia; 5 Faculty of Health Sciences, Curtin University, Bentley, Australia; 6 Telethon Kids Institute, Nedlands, Australia

**Keywords:** District health information systems 2, Solomon islands, Evaluation, Malaria, Reporting, Interview

## Abstract

**Background:**

District Health Information Systems 2 (DHIS2) is used for supporting health information management in 67 countries, including Solomon Islands. However, there have been few published evaluations of the performance of DHIS2-enhanced disease reporting systems, in particular for monitoring infectious diseases such as malaria. The aim of this study was to evaluate DHIS2 supported malaria reporting in Solomon Islands and to develop recommendations for improving the system.

**Methods:**

The evaluation was conducted in three administrative areas of Solomon Islands: Honoria City Council, and Malaita and Guadalcanal Provinces. Records of nine malaria indicators including report submission date, total malaria cases, *Plasmodium falciparum* case record, *Plasmodium vivax* case record, clinical malaria, malaria diagnosed with microscopy, malaria diagnosed with (rapid diagnostic test) (RDT), record of drug stocks and records of RDT stocks from 1st January to 31st December 2016 were extracted from the DHIS2 database. The indicators permitted assessment in four core areas: availability, completeness, timeliness and reliability. To explore perceptions and point of view of the stakeholders on the performance of the malaria case reporting system, focus group discussions were conducted with health centre nurses, whilst in-depth interviews were conducted with stakeholder representatives from government (province and national) staff and World Health Organization officials who were users of DHIS2.

**Results:**

Data were extracted from nine health centres in Honoria City Council and 64 health centres in Malaita Province. The completeness and timeliness from the two provinces of all nine indicators were 28.2% and 5.1%, respectively. The most reliable indicator in DHIS2 was ‘clinical malaria’ (i.e. numbers of clinically diagnosed malaria cases) with 62.4% reliability. Challenges to completeness were a lack of supervision, limited feedback, high workload, and a lack of training and refresher courses. Health centres located in geographically remote areas, a lack of regular transport, high workload and too many variables in the reporting forms led to delays in timely reporting. Reliability of reports was impacted by a lack of technical professionals such as statisticians and unavailability of tally sheets and reporting forms.

**Conclusion:**

The availability, completeness, timeliness and reliability of nine malaria indicators collected in DHIS2 were variable within the study area, but generally low. Continued onsite support, supervision, feedback and additional enhancements, such as electronic reporting will be required to further improve the malaria reporting system.

## Background

In 2018, an estimated 228 million cases of malaria occurred worldwide, with 405,000 deaths [1]. Malaria disproportionately affects children under 5 years, who accounted for up to 67% of all malaria deaths in 2018. Incidence of malaria cases and deaths in Solomon Islands is amongst the highest of all countries in the World Health Organization (WHO) Western Pacific region [2]. In 2018, there were 59,191 confirmed cases, of which 59.3% (35,072) were *Plasmodium vivax* and 26.7% (15,771) *Plasmodium falciparum*. There were also 109 deaths due to malaria [1]. *Anopheles farauti* is the primary vector and secondary vectors are *Anopheles punctulatus* and *Anopheles koliensis* [3, 4].

Malaria remains a significant cause of morbidity in Solomon Islands. Almost the entire population of Solomon Islands is at high risk for malaria, with only 1% of the population living in areas free of malaria. Between 1993 and 1999, control measures were decentralized to the provinces and were mainly based on the use of insecticide-treated bed nets (ITNs), house spraying using dichlorodiphenyltrichloroethane (DDT) and community awareness programs [5]. In 2003, long-lasting insecticidal nets (LLINs) were introduced in Solomon Islands [6]. Malaria is heterogenous across the provinces and, in 2008, Solomon Islands planned to eliminate malaria in two selected provinces by 2014: Isabel and Temotu [6, 7]. To support this national goal, case-based surveillance supported by a spatial decision support system (SDSS) was developed [8]. The SDSS was used for rapid case reporting and mapping, planning and deployment of preventive measures, including indoor residual spraying (IRS) and LLINs [9, 10]. However, use of the SDSS declined and cases have rebounded since then; notably, cases increased from 30,591 in 2015 to 86,343 in 2018 [1, 7]. In 2009, Solomon Islands along with nine other countries (Bhutan, China, Democratic People’s Republic of Korea, Indonesia, Malaysia, the Philippines, Republic of Korea, Sri Lanka, and Vanuatu; now expanded to 18 countries) established the Asia Pacific Malaria Elimination Network (APMEN), which aims to pursue the goal of eliminating malaria. Solomon Islands aims to eliminate malaria by 2030 [11].

**Fig. 1:**
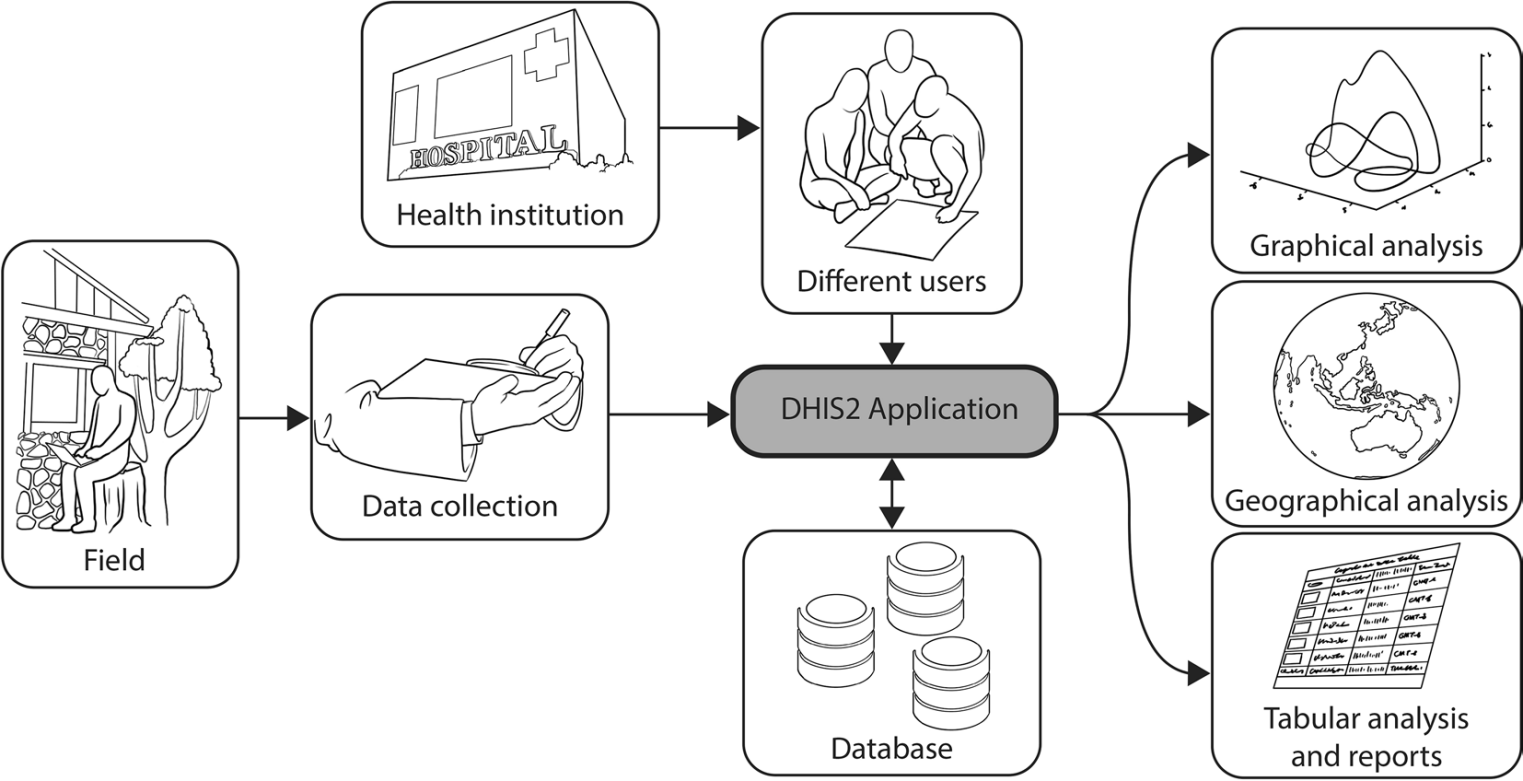
The functionality of District Health Information System 2 that is being used globally (Source: Adapted from Dehnavieh et al. 2018 [13])

Transforming malaria surveillance to become a core intervention strategy has been outlined in the *Global Technical Strategy for Malaria 2016–2030* (GTS) [12], and a key component is the improved use of data for decision-making. For health information management in resource-limited settings, WHO advocates the use of District Health Information Systems 2 (DHIS2). Since 1994, DHIS2 has evolved and is currently used in 67 countries for managing health information [13–16]. DHIS2 is an open-source information system with few hardware requirements and a flexible user interface that allows users to specify their content without the need for programming [17–19] (Fig. 1). DHIS2 is a generic tool that needs to be customized for local use for specific purposes such as disease surveillance.

DHIS2 provides a vehicle for standardization of data collection processes across public health facilities of a particular jurisdiction, usually a country. The data contained in DHIS2 can provide a useful means of establishing disease burden baselines that can be compared across districts and over time, allowing for assessment of community needs, intervention impacts and to evaluate the performance of public health programmes [20, 21]. In addition, DHIS2 has been used as a tool for integrated disease surveillance and response (IDSR) system and IDSR data have integrated into DHIS2 in sub-Saharan Africa [22, 23]. In Solomon Islands, DHIS2 is being used for infectious disease surveillance and management, and is the only health information management system that is currently being used for that purpose. A number of studies have outlined factors that limit the utilization and effectiveness of DHIS2. First, the integration of DHIS2 in national surveillance systems has been limited by a transient health workforce including public health staff and data managers [19]. Second, the limited availability of human resource capacity for data analytics has constrained the use of data that are collected [17, 24]. Thirdly, data that are collected but not used are an untapped resource, and processes are needed to realize the potential value of the data collated within DHIS2 to inform action. Fourthly, crucial infrastructure is often lacking, including internet reliability and coverage [15, 17, 25, 26]. However, there has been no specific evaluation of DHIS2 as a tool to support infectious disease surveillance. The aim of this study was to evaluate the performance of the DHIS2-enhanced malaria case-based reporting system in Solomon Islands using a mixed-methods approach and to provide evidence for improvements in the system to support effective decision-making.

## Methods

### Study site

**Fig. 2:**
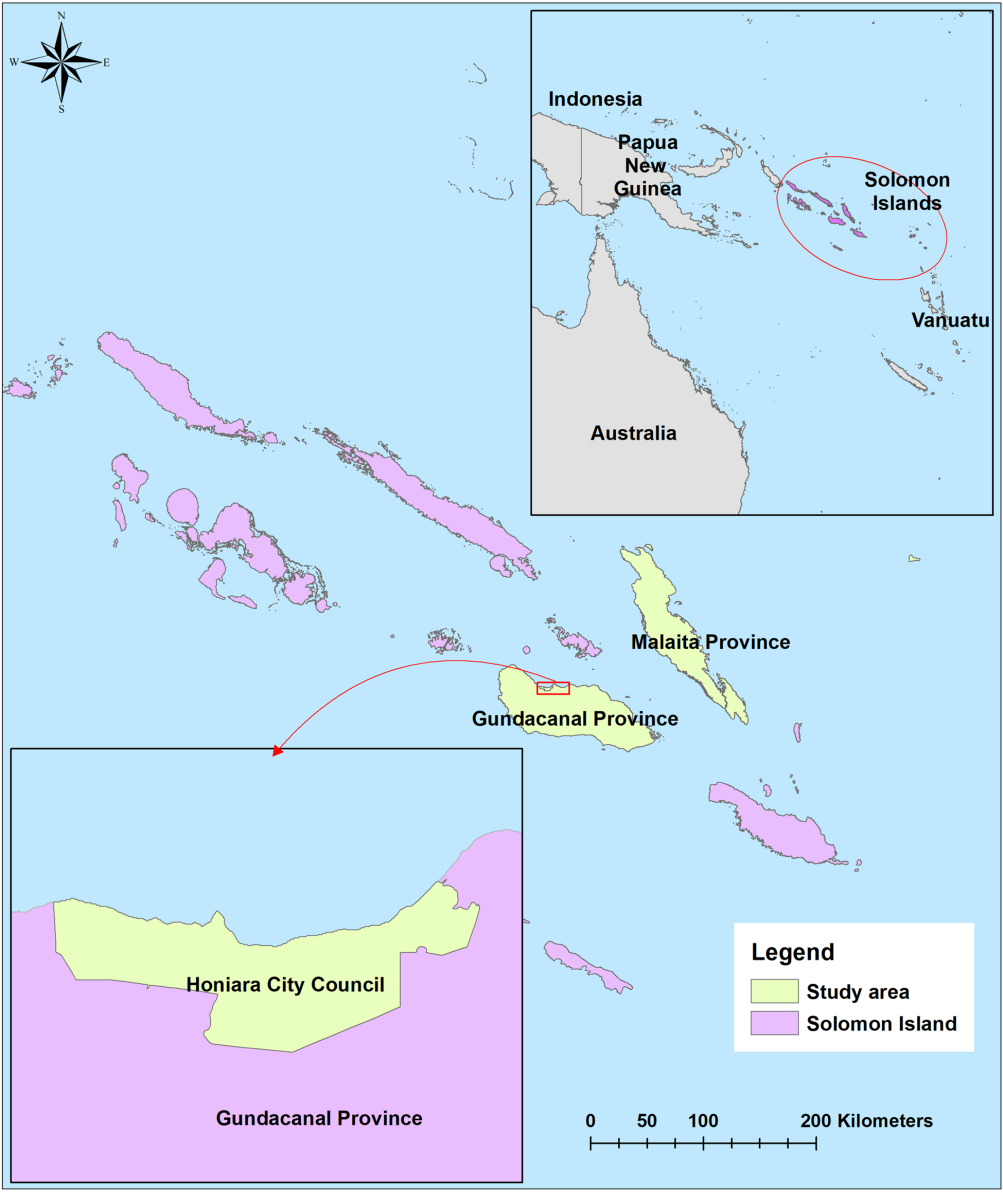
Map of Solomon Islands with study three study areas

Solomon Islands is a country located in the Melanesia sub-region of Oceania, with an estimated population of 667,044 in 2018 [27]. The country is administratively divided into nine provinces and Honoria City Council. Guadalcanal and Malaita provinces, and Honoria City Council, were selected for the current study because they report some of the highest numbers of malaria cases in the country, and they were relatively accessible to the study team (Fig. 2).

DHIS2 was introduced in Solomon Islands in 2015. As in other countries using DHIS2 around the world [28], malaria case-based data are extracted into a malaria case and morbidity reporting (MCMR) form every month at each health centre. The data come from various sources including outpatient department (OPD) records, microscopy, rapid diagnostic test (RDT) and stock registers and ledgers. These MCMR forms are sent to the provincial health office where the case-based data are entered into the online DHIS2. There is a system of zero reporting and the missing fields in MCMR are left as blank in DHIS2. The software provides options to generate quarterly or yearly reports by health centre or a range of administrative levels. The data stored in DHIS2 are available to registered users and other stakeholders through a password-protected log-in system.

### Data collection

Four core sets of criteria were used for the evaluation: availability, completeness, timeliness and reliability. Availability was defined as the presence or absence of malaria reporting forms at the provincial office. Completeness was defined as the percentage of observations within each of the nine indicators that were reported correctly [19, 29, 30]. Timeliness was defined as submitting reports to provincial offices prior to the 15th day of the subsequent month. Reliability was defined as the percentage of monthly reports that matched the records between MCMR forms and DHIS2 (Table 1). The nine indicators were: report submission date, total malaria cases, *P. falciparum* case record, *P. vivax* case record, clinical malaria, malaria diagnosed with microscopy, malaria diagnosed with RDT, record of drug stocks and records of RDT. These indicators were recommended by the Solomon Islands National Vector Borne Disease Control Program (NVBDCP) officials because of their importance for programme management. The details of the indicators are outlined in Table 2. However, report submission, drug stock and records of RDTs were not captured in DHIS2 so reliability was assessed for the remainder six indicators.

**Table 1:** Core areas and analysis plan for DHIS2 evaluation plan

Core areas	Indicators	Source*	Reference source^†^	Analysis
Availability	Presence/absence of monthly malaria report	Monthly malaria clinic reports	Not applicable	Presence/absence of monthly reports at province health departments
Completeness	Nine malaria indicators	MCMR	Not applicable	Proportion of completeness (total number of months with complete reporting in the selected health center/12 X number of health centres)
Timeliness	Nine malaria indicators	MCMR	Malaria clinics to provincial HIS	Time lag from the 15^th^ of the subsequent month from malaria clinics to provincial HIS
Reliability	Six malaria indicators	DHIS2	MCMR	Percent matching months between MCMR and DHIS2

*MCMR* malaria case and morbidity reporting form, *DHIS2* District Health Information systems 2, *HIS* Health Information System

*Source of data; ^†^Comparision group

**Table 2:** Nine malaria indicatiors extracted from malaria case and morbidity reporting form

No	Malaria indicators	Defination
	Report submission date	Date on which report was sent from health centre to provincial headquarters
	Total malaria cases	Total number of both *Plasmodium falciparum and P. vivax* cases treated in the health centre in a month
	*Plasmodium falciparum* case record	Total number of *P. falciparum* cases diagnosed and treated in the health centre in a month
	*Plasmodium vivax* case record	Total number of *P. vivax* cases diagnosed and treated in the health centre in a month
	Clinical malaria	Fever cases treated as malaria without blood test
	Malaria diagnosed with microscopy	Total malaria cases (both *P. falciparum* and *P. vivax*) diagnosed with microscopy in a month
	Malaria diagnosed with RDT	Total malaria cases (*P. falciparum)* diagnosed with microscopy in a month
	Record of drug stocks	Drug balance in the health centre at the end of month
	Records of RDT	RDT balance in the health centre at the end of month

A mixed-methods approach using quantitative and qualitative methods was used for this study. Mixed methods research can help address complex, multifaceted issues in health care delivery [31–34]. A qualitative study using focus group discussions (FGD) and in-depth interviews (IDI) was undertaken to elucidate the underlying reasons for the quantitative findings using a triangulation [35, 36]. Therefore, semi-structured questions focused mainly around the four themes outlined above. Quantitative and qualitative data were collected concurrently [37].

Honoria City Council, and Malaita and Guadalcanal Province were selected for convenience. Data were extracted from all nine health centres in Honoria City Council and 64 (out of 74) health centres’ available data for Malaita Province. The MCMR records were not available for Guadalcanal Province because these had been taken by Global Fund evaluators and had not yet been returned, and so the quantitative component of the study did not include Guadalcanal. Data pertaining to these indicators for the period 1st January to 31st December 2016 were extracted using a data extraction form. Results for each indicator were summarized by study province using means and percentages. Nine indicators in these four sets of criteria were extracted from the MCMR forms maintained at the respective provincial headquarters and DHIS2 because they were the only indicators that were captured in the MCMR forms.

Three FGD with the nurses (25) of Malaita and Guadalcanal provinces, and Honoria City Council, were conducted in June 2017. Thirteen IDI were run with key informants, also in June 2017. The FGD participants were purposively selected from the nurses of health centres who were involved in MCMR reporting, whilst the IDI participants were stakeholder representatives from government (province and national) and staff of the WHO country office who were working with DHIS2. FDG and IDI were undertaken to elicit opinions and feedback regarding the functionality and effectiveness of DHIS2 supported malaria reporting. During the interview, a flexible semistructure interview format was followed for both FGD and IDI. FGD lasted from 45 min to 1 h and IDI were conducted face to face and lasted from 30–45 min. The FDG participants express their opinions in orderly manner. The quotes were de-identified using an IDI code assigned by the man interviewer.

Unlike most investigators undertaking conventional qualitative data collection and analysis, the principal investigator did not have the opportunity to start data analysis from the very beginning of data collection, which can allow review of initial findings, identification of information gaps and tracking of data saturation [38]. Instead, all interviews were recorded in the first sitting and transcribed using professional transcriptors at a later date in Australia. A deductive coding method was used to identify major relevant themes relating to DHIS2 functionality and effectiveness. Pre-set coding schemes were formulated considering the study objectives, and were applied to the text. Qualitative data gathered from the field reports were analysed manually. The authors read and reread the transcripts and highlighted text under each of the main themes. Data were extracted and displayed using a matrix table, then interpreted under each of the main themes. Finally, the qualitative and quantitative analyses were correlated using triangulation to derive the study findings [39]. Quantitative data were extracted into Microsoft Excel (Microsoft Corp, Redmond, WA, USA) software and analysed using STATA version 16 (Stata Corporation, College Station, TX, USA). A Map was generated using ArcMap 10.5 software (ESRI, Redlands, CA).

## Results

From nine health centres in Honiara City Council, Bokona health centres reported the least records of total patients. Vura health centre treated most malaria cases and *P. falciparum*, while Pikinini clinic treated most *P. vivax* cases, Rove clinic treated most clinical cases of malaria as well as did most RDT tests and microscopy. Naha and Rove clinic had most RDTs and drugs in the stokes, respectively. Bokona health recorded the least cases and did the least tests.

Of 64 malaria clinics in Maliata Province, Auki clinic treated most cases of malaria, Atofifi clinic treated most *P. falciparum* and *P. vivax*, undertook microscopy. Rohinari did most tests using RDTs. Weiio treated the least *P. falciparum* and *P. vivax* cases, as well as clinical malaria. Similarly, Weiio clinic did the least tests with RDTs. Afenakwai, Arao, Darione, Sarawasi and Weiio did not report any *P. vivax*. Namolaelae clinic treated most clinical malaria while Weiio treated the least clinical cases. Many health centres did not report stock balance on RDT, while Rohinari had the most stocks. Nafinua had the most drug stocks.

A number of enablers and barriers to the DHIS2-enhance malaria reporting system were identified during the interview. Inadequate human resources was the common barrier affecting all the four themes. While the lack of regular transport and remote location impacted the timely reporting of MCMR. The high workload, lack training, feedback and supervisory visit was a common barrier to completeness, timeliness and reliability of reports. Availability and completeness affected by insufficient logistics, such as OPD, stock and laboratory registers, tally sheets and MCMR forms. Finally, lack of internet was a barrier for timely reporting and reliability of data.

### Availability

The source of data varied between health centres and the provincial office. OPD and laboratory registers, and stock ledgers were the source of data for the health centres. After provincial officials receive the MCMR forms, they enter the data into the online DHIS2 database. Microscopists usually undertook blood examination in health centres while RDTs were performed by nurses. Some of the facilities had shortage of RDTs and adequate number of OPD registers [“*(MCMR) book is there but sometimes need to photocopy extra copies for every table in OPD*” FDG3]. The results were extracted using MCMR forms by the nurses at the health centres and submitted to the provincial headquarters every month [“*[When] the patient comes to the health facility to see the nurses, nurses take their details and put them into the OPD register book in the clinic. At the end of the day they transfer the same information into the MCMR”* IDI4]. After MCMR forms reach the provincial headquarters, malaria monitoring and supervisor officers enter the data into the online DHIS2 software [“*So, my daily responsibility is for entering that (data) into the system (DHIS2)*” IDI9] (Table 3).

**Table 3:** Summary of factors associated with different evaluation themes of the DHIS2-enhanced malaria reporting system, Solomon Islands

Factors	Themes			
	Availability	Completeness	Timeliness	Reliability
Lack of transport			** + **	
Distance/ remote location			** + **	
Human resources	** + **	** + **	** + **	** + **
High workload		** + **	** + **	** + **
Logistics- register books*	** + **	** + **		
Lack of training		** + **	** + **	** + **
Lack of feedback		** + **	** + **	** + **
Lack of supervisory visits		** + **	** + **	** + **
Too many variables in MCMR form		** + **		
Lack of internet			** + **	** + **

+Factors related to the theme, *MCMR* malaria case and morbidity reporting form

*includes availability of OPD registers, stock register, laboratory record registers, tally sheets and MCMR forms

### Completeness

During the study period, completeness of all the nine indicator was 28%. The respondents had a good understanding of the completeness of reporting, including the need for regular reporting even when there were no cases to report [“ ‘*Forms completed’ means that each [form is] properly filled [with] no data missing,*” FGD1] and [“ ‘*completeness’ is filling all the things that are asked in the forms*” FGD2].

Nearly half (47.2%) of all indicators were complete in Honoria City Council, while only 25.5% were complete in Malaita Province. The highest completeness amongst the nine indicators was ‘*P. falciparum* diagnosed with RDT’ at 90.3%, followed by ‘*P. falciparum* and *P. vivax* diagnosed with a microscope’ at 90.1% each. The ‘stock balance of drugs’ and ‘stock balance of RDTs’ were the least complete indicators with 45.1 and 38.5% completeness respectively. Honoria City Council had better completeness than Malaita Province for all nine indicators. In Honoria City Council, ‘total parasites’, and ‘*P. falciparum* and *P. vivax* diagnosed with RDT’ were 95.4% complete. ‘RDT stock balance’ was the least complete indicator with only 53.8% complete. However, in Malaita Province, ‘*P. falciparum* diagnosed with microscopy’ was the most complete indicator, with 89.7% completeness, followed by ‘*P. falciparum* diagnosed with RDT’ with 89.6% completeness. Similar to Honoria City Council, ‘RDT stock balance’ was the least complete indicator with 36.5% completeness (Table 4).

**Table 4:** Completeness of nine indicators from malaria case and morbidity reporting forms, Solomon Islands

Indicators		Overall		HCC		MP	
		Number*	%	Number^†^	%	Number^‡^	%
Report submission date		559	63.8	86	79.6	473	61.6
Total treated cases		770	87.9	103	95.4	667	86.9
PF	RDT	791	90.3	103	95.4	688	89.6
PF	MIC	789	90.1	100	92.6	689	89.7
PV	MIC	789	90.1	103	95.4	686	89.3
Clinical malaria		782	89.3	103	95.4	679	88.4
Total test	MIC	499	57.0	91	84.3	408	53.1
Total test	RDT	611	69.8	89	82.4	522	68.0
Drug stock		395	45.1	71	65.7	324	41.2
RDT stock		337	38.5	57	52.8	280	36.5

*HCC* Honiara City Council, *MP* Malaita Province, *PF* *Plasmodium falciparum*, *PV* *P. vivax;* *RDT* rapid diagnostic test, MIC microscopy

*Expected number (number of health centres X 12) = 876; ^†^Expected number = 108; ^‡^Expected number = 768

Several factors facilitated the completeness of reporting. Nurses were assisted in filling in all fields of the reporting forms by other staff members in the health centre, such as microscopists and laboratory technicians. As they shared the same office premises, nurses sought the help of microscopist and laboratory technicians when they needed clarification on the data recorded by them. Other enablers included training and supervision of nurses by the provincial supervisors, [“*There should be … constant refresher [and] training in how the data should be documented because … those are the things [that] can improve [completeness]*” IDI10].

The study respondents also identified some challenges for the completeness of reporting. The handwriting of nurses was often not clear enough to understand the information written in the registers. Heavy workload critically affected the completeness of reporting, [“*There is a heavy workload, lots of times, …. there is lots of pressure, demand from patients and we run all over the place and in the end we forgot to write some [information] of these people in the record book*” FGD1]. As a result of a heavy workload, nurses often multi-tasked, including providing regular care to the clinic patients whilst attending to administrative matters such as recording in the registers [“.. *talking from my experience when a clinic does not have too many patients, we pay attention when filling up the forms. But when we have lots of patients and you are alone, that’s the problem*” FGD2]. A lack of regular training and refresher courses also led to incomplete forms, [“*In our unit, some of the staff [nurses] did not attend the training. So they lack the understanding of the filling up of forms [correctly]*” FDG1], and *[“[Provinical] officials should come and give us training. See that we are doing the right thing, [we] understand [filling] the form properly, then we will be able to [properly fill the forms]*” FDG2]. The workload associated with reporting the individual records of all patients was seen as one of the reasons for incomplete reporting. This was true especially in some health centres with a large number of cases. In this regards, a participant in an IDI said, “…*for a small country like Solomon [Islands]… entering 86,000 cases … is a huge task. … for the some of the provinces you’ll need at least two or three people just doing only this”* [IDI6].

Use of technology such as computers that can automatically summarize the records for the month at the provincial level (as opposed to the health centre level, where DHIS2 is used to complete this task) can help improve the completeness of reporting, [“ *I feel I should have a computer or programme that I fill in the data here and it summarizes the data at the end of the month …[so that] I don’t have to do [a] tally*” FDG2]. The nurses thought that supervisory visits also helped in improving the completeness of reporting. During those visits, a supervisor would have the opportunity to review the draft report and provide feedback to the nurses if they find any incomplete or inconsistency reporting. However, regular supervisory visits were not often observed in all areas due to inadequate funding and availability of supervisory level staff members [“*[without supervisory visits] that’s when nurses don’t think seriously about the importance of these quality data collection because, like those at a higher level, they didn’t come down and visit us to give feedback, supervision. We take this information for granted*” FGD2] (Table 3).

### Timeliness

**Fig. 3:**
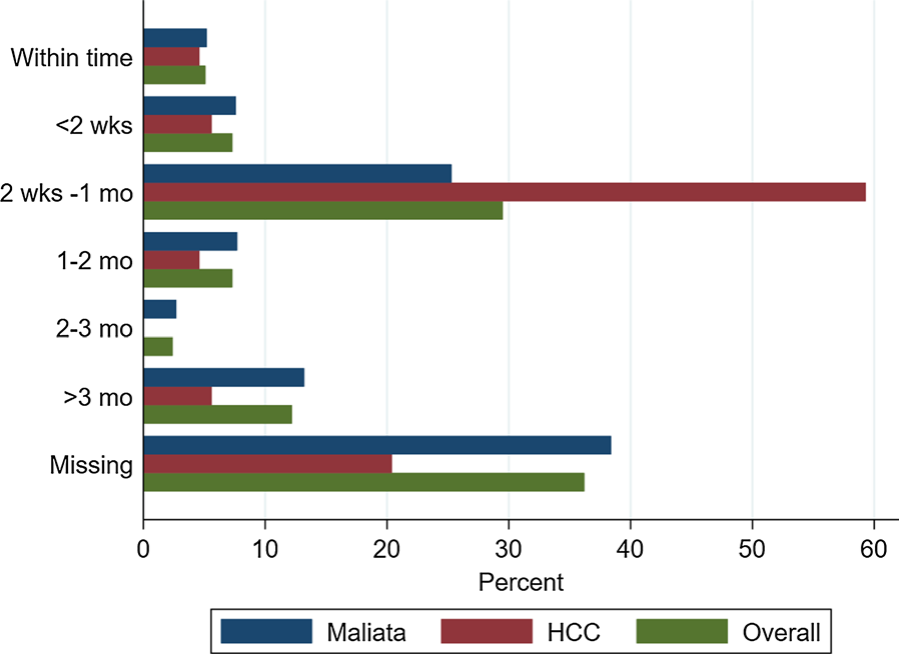
Timeliness of reporting from malaria clinics to the provincial information office of Malaita province, HCC and overall. *HCC* Honiara City Council

The interview respondents knew that the reports should reach the provincial headquarters by the 15th of the following month [“*From the province, they set up a timely reporting period for us if we submit before first two weeks or 15th of each month, then we are timely reporting*” FGD2]. However, there was a significant delay in submitting reports, with only 5.1% (45/976) of health centre-months submitting reports before the 15th of the subsequent month. The submission date was not recorded on 36.2% (317/876) of MCMR forms, making it difficult to determine the timeliness of these reports. The most common lag time of the reports was 2 weeks to 1 month (29.5%, 258/876). Honiara City Council received around 60% of reports being transferred within the range of two-weeks to one month as opposed to 25% for Malaita Province (Fig. 3). Despite the quantitative findings, study participants felt that there was timely reporting, [“*The timeliness of reporting for the province is more than 80%*” IDI10]. As for DHIS2 data entry at the province, NVBDCP set a deadline that all data of a calendar month should be entered by four weeks of the following month, giving the provinces two extra weeks to enter the data from the date of receipt of data from the health centres (assuming data were submitted on time).

The FGD and IDI participants outlined many challenges to timely reporting. The use of physical reporting forms that needed to be transported to provincial offices meant that the remoteness of some health centres impeded timely reporting [“S*ome of the clinics are far like in the southern region, they could not do reporting on time*” FGD1]. A respondent in an IDI said, [“*It’s a bit hard because some clinics or health facilities … are remote…… far away so that’s why the reports are coming in late*” IDI2]. The MCMR report was ready in health centres but due to a lack of transportation facilities, they could not send the report to the provincial headquarters.

Other challenges included workload, inadequate infrastructure facilities such as separate offices, computers, uninterrupted internet access and communication systems to collect reports from geographically remote health facilities. The workload issues were raised by several respondents, [“*Yes, [because of] workload …. Sometimes they don’t have time [to submit the report]*” IDI8].

In addition, they had to input too many variables in the reporting forms, “…..*sometimes it is difficult to fill a variety of information into the form. … we found out [it] is time-consuming especially in the bigger health facilities”* FGD1]. Besides malaria, nurses were involved in reporting to other programs and had to use several reporting forms. Finally, a lack of regular supervision and feedback affected timely reporting [“*…. lack of feedback and supervision [means] we are not motivated to submit timely reporting*” FGD3].

Study participants suggested ways of improving timeliness of reporting. They suggested good transportation facilities are critical to timely reporting [“*We can improve the late reporting through improving transport*” IDI2]. In areas where the transportation facilities were erratic, it was proposed that the provincial supervisor officer could collect the reports by visiting these health centres. Communication technologies can enhance timely reporting, for instance, telephones can be used to collect reports from remote health centres. In some areas, they also used two-ways radio for communication. However, budgets often limited use of these communication methods (Table 3).

### Reliability

Information about the date of report submission, RDT and drug balance are not captured in DHIS2 despite these items being reported in the MCMR. The most reliable indicator in DHIS2 was ‘clinical malaria’ with 52.2% reliability followed by ‘*P. falciparum* diagnosed with microscopy’ at 48.4% reliability. The least reliable indicator was ‘*P. vivax* diagnosed with RDT’ at 29.9% reliability. Data from Malaita Province had higher reliability as compared to Honoria City Council. In Honoria City Council, the most reliable indicator was ‘clinical malaria’ at 67.6% reliability and the least reliable indicator was the ‘total tested with microscopy’ with 20.4% reliability. In Malaita Province, the most reliable indicator was ‘clinical malaria’ at 50.0% reliability and the least reliable was ‘*P. falciparum* diagnosed with RDT’ at 30.2% reliability (Table 5).

**Table 5:** Reliability of six indicators in DHIS2 as compared to MCMR

Indicators^*^		Overall		HCC		MP	
		Number	%	Number	%	Number	%
Total parasites		334	38.1	38	35.2	296	38.5
PF	RDT	262	29.9	30	27.8	232	30.2
PV	MIC	424	48.4	44	40.7	380	49.5
Clinical malaria		457	52.2	73	67.6	384	50.0
Total test	MIC	361	41.2	22	20.4	339	44.1
Total test	RDT	296	33.8	24	22.2	272	34.4

*HCC* Honiara City Council, *MP* Malaita Province, *PF* *Plasmodium falciparum*, *PV* *P. vivax*, *RDT* rapid diagnostic test, *MIC* microscopy

*Three indicators namely report submission date, drug and RDT balance were not recorded in online DHIS2 database

The interview respondents recognized that reliability of the malaria case reporting system meant that the data should be accurate. The data should be consistent with the other variables in the form as well as when matched with the different data sources (i.e. OPD registers and RDT and microscopy record books). Some respondents reported that there might have been some variance between the data sources, but this should be minimal [“*Of course there’s a bit of variance there, which you have to expect. You never get 100%*”].

The respondents identified multiple challenges in reliability in malaria case reporting. Similar to availability and completeness, inadequate human resources affected data reliability. The local and tertiary-level health facilities were unable to verify or assess the quality of data [“…*unfortunately, the medical statistics unit doesn’t have the human resources capacity. They have just four coordinators, even out of the four coordinators two have left- they are only left with one now*” IDI6]. Availability of adequate supervisory-level staff members was critical, particularly to assess data quality on a regular basis.

The respondents linked data reliability to the availability of adequate logistics. There were some facilities with a scarcity of tally sheets and reporting forms *[“…when the forms run out, [we are] not able to submit the reports*” FGD2]. Another factor that affects the reliability of the data is programs frequently changing their forms, driven by donors who want to collect data on new indicators. Inadequate and inconsistent internet facilities were one of the main limitations of online DHIS2 reporting. Generally speaking, there were not enough officials to enter data into the online DHIS2 database, there were not enough computers and due to insufficient internet connectivity, the provincial surveillance office delayed entry of the data, leading to limited time to assess and verify the data.

The respondents made several recommendations to improve data reliability. Use of devices, such as computers, mobile phones or tablets, for data collection could address several reliability-related challenges [“*…we don’t have computers and everything is manual that’s why it is challenging*” FGD3]. These devices would not only help in data collection but also enable the staff members to connect with their supervisors when they need instructions or guidance on reporting, especially when filling out forms. Arranging refresher training at regular intervals may help staff members to ensure quality data collection and reporting. Increased use of the DHIS2 data system could help in improving data reliability. For example, if data from the system were used for policy decision-making regularly by government authorities, errors would be more likely to be identified and fixed, with an increased investment to ensure adequate human resources, infrastructure and facilities. A respondent in an IDI said, “*The best way to improve reliability is to improve the demand for their time. The more the data is used the more issues that they will encounter with the data, the more ways you will find out to actually fix these issues”* [IDI6] (Table 3).

## Discussion

This study identified significant performance issues with the malaria case reporting system in Solomon Islands. Notably, the completeness of data ranged from 38.5 to 90.3% and only 5.1% of reports were submitted on time (i.e. before the 15th of the subsequent month). The highest percentage of reliability for any indicator was only 62.4%. The main challenges for completeness were a lack of supervision, limited feedback, inadequate human resources, a lack of training and refresher courses and the high workload burden associated with case-based reporting. Enablers of improved system performance would include regular supervisory visits and training and provision of computers for recording data at the health centres. Challenges of timeliness were the remote locations of some health centres, a lack of regular transportation, inadequate human resources and too many variables in the reporting forms. These challenges can be addressed by the use of alternative communication technologies such as phones and two-way radio. The main challenges to reliability were a lack of trained technical professionals such as statisticians, donor-driven data collection forms, and unavailability of items such as tally sheets and reporting forms. This can be addressed through training, and provision of computers, mobile phones or tablets for data collection.

Computerisation of data collection, analysis and data transfer is often offered as the answer to health information problems [40]. This was consistently highlighted in this study where participants recommended moving from a paper-based system to technology-based data recording and reporting. The benefits of using such technologies can be obtained at different levels of health systems. In health centres, they can help in reducing the workload, and improve data collection and reporting [41, 42]. At the district, provincial and national level, they can enhance monitoring, support supervision and feedback [41]. However, uptake of technology-based recording and reporting is hampered by a lack of awareness and organizational support [34, 43]. Other barriers include inadequate internet coverage [31, 44], telecommunications network coverage [42] and electricity [45], limited budgets for system introduction, maintenance and repair of devices [46–48] and limited training of personnel in their use [49, 50]. The expansion of computerized recording and reporting in Solomon Islands need to address these barriers for expanding it to the health centres.

Notably, the completeness of reporting in this study was better than findings from Kenya and Nigeria [19, 30]. The latter two studies were conducted in settings with more intensive transmission and where public health programmes focus on malaria control rather than elimination. In Solomon Islands, transmission is more focal and case-based reporting is done to help achieve the goal of malaria elimination [51]. In elimination settings, data completeness is particularly important, as the system needs to identify all cases of malaria.

Completeness was less than 50% for reporting of anti-malarial drugs and RDTs in the health centres. This is concerning because health centres in Solomon Islands are located in remote areas with limited transportation. These indicators are important because they help prevent shortage of drugs and RDTs in hard-to-reach health centres. Therefore, it is important to take measures to increase the completeness of these two useful indicators. Completeness of reporting can be increased through the provision of additional training, in the form of refresher courses, as suggested by participants in this and other studies [19, 52]. The reporting quality greatly improved following training on data use in Tanzania [53]. Another area that needs urgent attention both at the level of health centres and provinces pertains to the shortage of human resources. This will require long-term planning and investment.

The national policy of the Ministry of Health and Medical Services of Solomon Islands requires all health centres to submit their monthly report to the provincial health centre within two weeks, defined as the 15th day of the subsequent month. Most of the participants understood this timeline of report submission. However, only 5.1% of cases were reported within the reporting time and 36.2% did not have the submission date recorded on the forms. This is much lower than a report from Uganda, where timeliness of outpatient reporting was 22.4% in 2011–2012 and increased to 85.3% in 2012–2013 [24]. Among other reasons, the current system is a two-tier system of paper-based reporting and entry into the electronic DHIS2 database, which seems to be impacting on timely reporting. In two districts of Zambia a successful pilot has been reported, whereby mobile phones applications are used to submit reports including cases, and medical and diagnostics supplies to a DHIS2 database, and from which feed-back is received by field officers [54]. Other studies have shown electronic reporting can be effective tools for submitting reports in near real-time [55–57]. Unlike other programs such as for tuberculosis, acute respiratory infections and immunization that involve aggregated reporting, the malaria program in Solomon Islands uses case-based reporting. This requires considerable time for data extraction from registers such as OPD, microscopy, RDT and stock registers [58]. As a result, there were occasions where other program reports (which involved aggregate reporting) arrived on time while malaria reports were submitted late. A tradeoff between the number of variables and the time available to undertake the work should be considered so that the quality of the reported data is not impacted [58]. In order to provide adequate time, reporting time could be extended from the 15th to the 30th of the subsequent month as a more realistic timeline, although this will result in delays in identifying emerging patterns of malaria. Alternatively, data entry into DHIS2 using phones or computers could be extended to health centres. Experiences from other countries using electronic reporting showed that all facets of reporting including timeliness, completeness and ease of reporting improved [40–42, 54, 59, 60]. A higher percentage of reports were submitted within the required timeframe from health centres in Honiara City Council as compared to Malaita Province. This could be due to better transportation facilities in Honiara City Council and the remoteness of Malaita Province.

Reliability of MCMR reports and those in the DHIS2 database varied considerably. The reliability of malaria reporting was computed by comparing information in the MCMR and DHIS2 and did not include a full data audit from the health centres. Reliability of all the nine indicators could not be established because three indicators, namely date of report submission, RDT use and drug balance, were not recorded in the online DHIS2 database. Ideally, the DHIS2 database should be modified to include this information. DHIS2 should be a single, comprehensive database and data visualisation tool that enables different sources of information to be accessed and analysed concurrently. Studies have shown that having a multiple reporting systems with limited coordination give rise to redundancies and wastage of resources [61]. Data reliability in Solomon Islands can be improved if health officials are provided with basic statistical training, rather than posting a statistician.

Malaita province demonstrated higher reliability than Honiara City Council in most indicators possibly due to higher cases in each health centre in the latter. An earlier study in Solomon Islands showed that the mean data discrepancy between the malaria records of health centres and government statistics was 21.2% [62]. The reasons were high numbers of patients leading to over work load, illegible writing, the disuse of tally sheets, and insufficient resources at some health centres. Whilst not conducted in the current study, a full data audit in selected health centres would have provided information on levels of consistency between the MCMR and primary data sources, such as OPD, laboratory and stock registers.

In most developing countries, the process of collection, collation, compilation, analysis and reporting of health care data is limited by inadequate human resources- both in terms of skills and required numbers. Additionally, a lack of data ownership compounded by health workers’ perception that the purpose of a health information system is simply to enable submission of reports to the higher levels, leads to a situation where there is no incentive for health workers at levels below the national level to analyse, use and interpret health data [17, 24, 63]. This results in poor quality data, as identified in our study and other studies in developing countries [64, 65]. However, reliable information when generated by manual or technological systems is important for quality decision-making, which ultimately leads to improved quality of care [66]. Conversely, inaccurate information can lead to poor choices in health investments. Regular feedback to the health centre is also important in improving the quality of data [36, 64, 67]. Feedback is a form of training and directly addresses the causes of poor quality data and enhances awareness of the importance of data. However, a lack of regular feedback from the province to the health centres occurred in our study, which was due to inadequate human resources at the province level. The government of Solomon Islands needs to address this issue in earnest [68].

Some of the problems highlighted above can be addressed by supporting electronic data capture from health centres [53]. The formerly used SDSS was successful in enabling the digital capture of data from remote parts of Solomon Islands [8–10] and Bhutan [57]. The SDSS in Solomon Islands was initially developed to support frontline intervention management as part of donor-funded provincial-based malaria elimination programmes (notably those in Isabel and Temotu Province) [10]. Following the completion of these campaigns and the successful implementation of SDSS to specifically support IRS, LLIN distribution and case-based surveillance response interventions in these elimination programmes, a lack of continued resources were provided to further adapt SDSS applications to support the broader malaria control programme- despite internal programme interest to do so. Plans however do remain to further progress SDSS-based targeted support in high priority locations pending future external support and resource allocation to enable this. Extension of data entry to health centres is now possible with the DHIS2 enhanced system [69, 70] because DHIS2 has developed an android app which allows for an offline data entry [71]. More work needs to be done to explore this and other technological solutions for improving DHIS2 applications for malaria and other infectious diseases.

## Limitations and strengths of the study

This study is subjected to a number of limitations and strengths. Firstly, this study was undertaken in two provinces and the Honiara City Council, and the findings may not represent those of other provinces or the national situation. Secondly, the non-random sampling of study sites limits our ability to generalize the results more widely. Thirdly, resources were not available for double extraction and double-entry when extracting data from the MCMR forms. Finally, DHIS2 software was introduced only two years before the study and the reporting may have improved since then. The FGDs and IDIs were conducted in one field trip and we did not have the opportunity to review the interview approach to identify gaps and then refine the approach in subsequent interviews. Additionally, study researchers were unable to assess data saturation during data collection that may enable measurement of the richness of the qualitative findings. Failure to reach data saturation may have hampered content validity [72]. Despite these limitations, a major strength of the study was the use of multiple data sources to clarify each of the main themes. This provided a richness of information which, upon analysis within and across the data sources, enabled the discovery of a number of consistencies as well as diversity in the findings.

## Conclusion

The availability, completeness, timeliness and reliability of nine malaria indicators collected in DHIS2 were variable within the two study sites where this information was available but generally low. Extension of electronic data capture to health centres would improve the timeliness, completeness and reliability of reporting. Continued onsite support, supervision, feedback and additional system (especially infrastructure) enhancements, such computers will be required to further increase completeness and reliability of the reports.

## Data Availability

Data are available upon reasonable request by an email to the corresponding author.
